# Pre-asthma: a useful concept for prevention and disease-modification? A EUFOREA paper. Part 1—allergic asthma

**DOI:** 10.3389/falgy.2023.1291185

**Published:** 2024-01-30

**Authors:** G. K. Scadding, M. McDonald, V. Backer, G. Scadding, M. Bernal-Sprekelsen, D. M. Conti, E. De Corso, Z. Diamant, C. Gray, C. Hopkins, M. Jesenak, P. Johansen, J. Kappen, J. Mullol, D. Price, S. Quirce, S. Reitsma, S. Salmi, B. Senior, J. P. Thyssen, U. Wahn, P. W. Hellings

**Affiliations:** ^1^ Department of Allergy & Rhinology, Royal National ENT Hospital, London, United Kingdom; ^2^ Division of Immunity and Infection, University College, London, United Kingdom; ^3^ The Allergy Clinic, Blairgowrie, Randburg, South Africa; ^4^ Department of Otorhinolaryngology, Head & Neck Surgery, and Audiology, Rigshospitalet, Copenhagen University, Copenhagen, Denmark; ^5^ Allergy, Royal Brompton Hospital, London, United Kingdom; ^6^ Head of ORL-Deptartment, Clinic Barcelona, Barcelona, Spain; ^7^ Chair of ORL, University of Barcelona, Barcelona, Spain; ^8^ The European Forum for Research and Education in Allergy and Airway Diseases Scientific Expert Team Members, Brussels, Belgium; ^9^ Otolaryngology Head and Neck Surgery, A. Gemelli University Hospital Foundation IRCCS, Rome, Italy; ^10^ Department of Respiratory Medicine & Allergology, Institute for Clinical Science, Skane University Hospital, Lund University, Lund, Sweden; ^11^ Department of Respiratory Medicine, First Faculty of Medicine, Charles University and Thomayer Hospital, Prague, Czech Republic; ^12^ Department Clinical Pharmacy and Pharmacology, University of Groningen, University Medical Center Groningen, Groningen, Netherlands; ^13^ Deptarment of Microbiology Immunology & Transplantation, KU Leuven, Catholic University of Leuven, Leuven, Belgium; ^14^ Paediatric Allergist, Red Cross Children’s Hospital and University of Cape Town, Cape Town, South Africa; ^15^ Kidsallergy Centre, Cape Town, South Africa; ^16^ Department of Rhinology and Skull Base Surgery, Guy’s and St Thomas’ Hospital NHS Foundation Trust, London, United Kingdom; ^17^ Department of Clinical Immunology and Allergology, University Teaching Hospital in Martin, Martin, Slovakia; ^18^ Department of Paediatrics, Jessenius Faculty of Medicine in Martin, Comenius University in Bratislava, University Teaching Hospital in Martin, Martin, Slovakia; ^19^ Department of Pulmonology and Phthisiology, Jessenius Faculty of Medicine in Martin, Comenius University in Bratislava, University Teaching Hospital in Martin, Martin, Slovakia; ^20^ Department of Dermatology, University of Zurich, Zurich, Switzerland; ^21^ Department of Dermatology, University Hospital of Zurich, Zurich, Switzerland; ^22^ Department of Pulmonology, STZ Centre of Excellence for Asthma, COPD and Respiratory Allergy, Franciscus Gasthuis & Vlietland, Rotterdam, Netherlands; ^23^ Rhinology Unit and Smell Clinic, ENT Department, Hospital Clínic, FRCB-IDIBAPS, Universitat de Barcelona, CIBERES, Barcelona, Spain; ^24^ Observational and Pragmatic Research Institute, Singapore, Singapore; ^25^ Division of Applied Health Sciences, Centre of Academic Primary Care, University of Aberdeen, Aberdeen, United Kingdom; ^26^ Department of Allergy, La Paz University Hospital, IdiPAZ, Madrid, Spain; ^27^ Department of Otorhinolarynogology and Head/Neck Surgery, Amsterdam University Medical Centres, Location AMC, University of Amsterdam, Amsterdam, Netherlands; ^28^ Department of Otorhinolaryngology, Kuopio University Hospital and University of Eastern Finland, Kuopio, Finland; ^29^ Department of Allergy, Inflammation Center, Helsinki University Hospital and University of Helsinki, Helsinki, Finland; ^30^ Department of Otolaryngology/Head and Neck Surgery, University of North Carolina at Chapel Hill, Chapel Hill, NC, United States; ^31^ Department of Dermatology, Bispebjerg Hospital, University of Copenhagen, Copenhagen, Denmark; ^32^ Former Head of the Department for Pediatric Pneumology and Immunology, Charite University Medicine, Berlin, Germany; ^33^ Department of Otorhinolaryngology-Head and Neck Surgery, University Hospitals, Leuven, Belgium; ^34^ Laboratory of Allergy and Clinical Immunology, University Hospitals Leuven, Leuven, Belgium; ^35^ Upper Airways Research Laboratory, Department of Head and Skin, Ghent University, Ghent, Belgium

**Keywords:** pre-asthma, asthma, quality of life, asthma natural history, predisposition, risk factors

## Abstract

Asthma, which affects some 300 million people worldwide and caused 455,000 deaths in 2019, is a significant burden to suffers and to society. It is the most common chronic disease in children and represents one of the major causes for years lived with disability. Significant efforts are made by organizations such as WHO in improving the diagnosis, treatment and monitoring of asthma. However asthma prevention has been less studied. Currently there is a concept of pre- diabetes which allows a reduction in full blown diabetes if diet and exercise are undertaken. Similar predictive states are found in Alzheimer's and Parkinson's diseases. In this paper we explore the possibilities for asthma prevention, both at population level and also investigate the possibility of defining a state of pre-asthma, in which intensive treatment could reduce progression to asthma. Since asthma is a heterogeneous condition, this paper is concerned with allergic asthma. A subsequent one will deal with late onset eosinophilic asthma.

## 1 Introduction

Several chronic diseases only become symptomatic from the moment a certain threshold of inflammation or volume of tissue damage has been reached (1–4). By this stage the disease may be irreversible with no possibility of cure (1, 5). Recently the idea of earlier pre-emptive diagnosis has evolved. For example, the concept of pre-diabetes has led to identification of likely future diabetics and provision of measures to reduce disease progression. Similarly, the pre-diagnostic phase of Alzheimer's and Parkinson's diseases are being explored in population-based studies.

In this paper we explore the possibilities for similar action in asthma.

### 1.1 Pre- diabetes. An example of early intervention to reduce disease progression

The concept of pre- diabetes, a state of intermediate hyperglycemia of which the sufferer is unaware, with a significant risk for diabetes development and associated adverse health outcomes, has proved useful. Lifestyle advice has been shown in studies to lead to a 58% risk reduction in diabetes, improving health, reducing some morbidities and costs (6). Weight loss was the biggest determinant of risk reduction: with a 16% decrease in diabetes development for every kilogram lost (7). As pre- diabetes affects some 352 million adults between the ages of 20 and 79 (7.3% of that population) and health costs for those with diabetes are double those of people without it, this is cost-effective preventive medicine (8, 9).

The soluble oligomer binding assay (SOBA), detects toxic (alpha sheet) Aβ oligomers in serum long before symptoms of Alzheimer's disease emerge. This should enable earlier diagnosis and raises the possibility of prevention of full -blown neurodegeneration (10).

Parkinson's, a disease caused by a lack of dopamine, is often present for several years before the diagnosis is made. Machine learning applied to voice recordings can be used to detect Parkinson's disease automatically and early, allowing earlier treatment and better quality of life for sufferers (11).

### 1.2 Asthma

Asthma is a highly prevalent disease of modern societies, affecting 1–29% of the population of different countries, some 3–400 million individuals being affected worldwide (12, 13).

Asthma is occasionally fatal; in most patients it reduces quality of life (QoL), requires long term daily treatment and is costly to the individuals, their families and to society. A significant number of people with severe asthma receive long-term oral corticosteroid (OCS) treatment, which can result in adverse outcomes and increase long-term healthcare costs (14). Recently more widely available monoclonal antibodies provide an alternative option for some severely affected patients but are extremely expensive.

At present, several risk factors for asthma are universally recognised, yet there is a lack of routinely applied screening programmes for asthma in individuals at risk. A recent systematic review of childhood asthma predictive tools concluded that they have” poor predictive accuracy with performance variation in sensitivity and positive predictive value” (15).

Is there a possibility of defining a state of pre- asthma whereby preventive measures could be taken to reduce asthma development? In this paper we consider what is currently known about the origins and pathophysiology of asthma in order to explore the potential for its prevention both at population level and if pre-asthma could be identified.

Search terms including” asthma” together with” causes”, “phenotypes”, “endotypes”, “genomics”, “origins”, “natural history”, “pathogenesis”,” predisposition “, “provoking factors” were used, plus “allergic march” together with the Global Initiative for Asthma (GINA) and the British Thoracic Society/Scottish Intercollegiate Guidelines Network (BTS/SIGN) guidelines, references contained within the articles found plus articles in the collections of the authors.

## 2 What is asthma?

Asthma is a chronic lung disease affecting people of all ages. It is caused by inflammation and muscle tightening around the airways, which makes it harder to breathe (16). Clinically asthma is characterized by a symptom complex of wheeze, shortness of breath, cough and chest tightness together with reduction in expiratory outflow which is initially variable but later may become fixed.

### 2.1 How does asthma arise?

**Figure 1:**
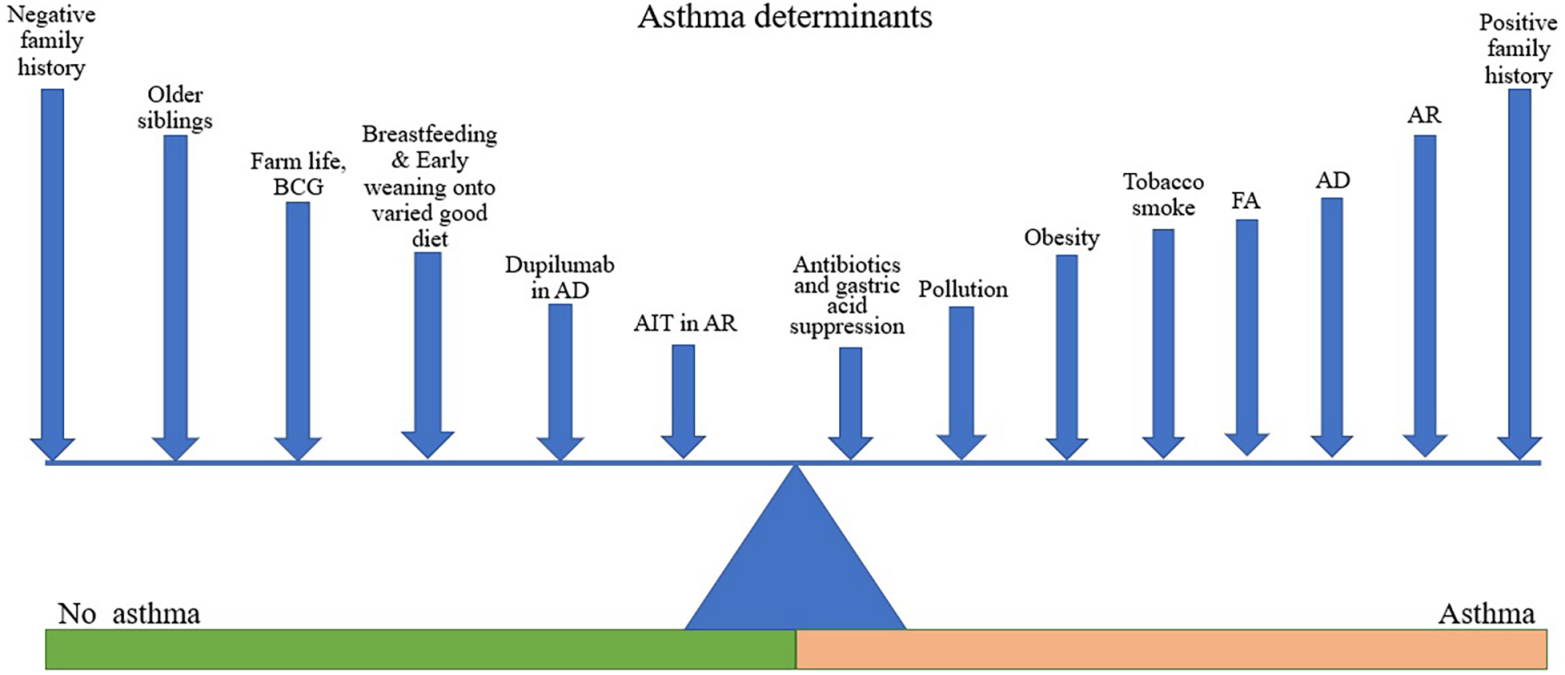
Asthma determinants. Figure shows various factors acting to promote or reduce the likelihood of asthma development. The length of line is not intended to denote the magnitude of effect but is varied to facilitate labelling. Different factors may be more or less relevant in different individuals depending on their genetic background.

Asthma is a heterogeneous condition with various phenotypes and endotypes usually associated with chronic airways inflammation and hyperresponsiveness, though rarer non-inflammatory subtypes exist (17). It is a variable disease in which symptoms and signs can be precipitated by physical and environmental triggers, including stress and exercise, weather or temperature changes, smoke, airborne particles and chemicals, microbes (mainly viruses), and allergens; several of these acting initially on the respiratory epithelium (18, 19) (Figure 1). The genetic background of the individual affects the response which may also be modified by exercise (20), thus both hereditary and epigenetic factors are at play in asthma pathogenesis.

The main drivers of inflammation include allergens, viruses and pollutants in allergic asthma, in other forms they are less well identified. Based on inflammatory profile we can distinguish two major inflammatory forms of asthma, both of which are usually associated with Type 2 eosinophilic inflammation: allergic asthma and late-onset (adult) asthma, which is mainly non- allergic. In children allergic asthma predominates and asthma incidence and prevalence are higher; morbidity and mortality are higher in adults (21).

This paper will focus on allergic asthma in childhood since this form of asthma has a well—described pathophysiology and known triggers.

#### 2.1.1 Substances acting on the epithelium

##### 2.1.1.1 Allergens

A fundamental property of the immune system is its ability to distinguish between “self” and “non-self”, that is, between molecules produced by the “self”s” own genes and those that are not. In atopic asthma, allergens release proteases, which disrupt the epithelial barrier and induce secretion of alarmins such as interleukin-25 (IL-25), interleukin-33 (IL-33), and thymic stromal lymphopoietin (TSLP) from epithelial cells. IL-33 and TSLP activate dendritic cells (DCs) which then present antigens to naïve T cells, which are activated to differentiate into Th2 cells. Th2 cells, together with alarmin-stimulated IL-C2 innate immune cells which are largely located at epithelial surfaces (22), are the key cellular mediators of allergic airway inflammation.

##### 2.1.1.2 Air pollutants

###### 2.1.1.2.1 Tobacco smoke

People who smoke tobacco are exposed to nicotine and a range of combustion products such as carbon particles, partially combusted hydrocarbons, lipopolysaccharides, and a range of gases. Smokers with existing asthma have increased morbidity, more severe asthma symptoms, accelerated decline in lung function, reduced lung growth, an altered inflammatory phenotype, and reduced corticosteroid responsiveness with increasing neutrophilic inflammation. Furthermore, smoking introduces several changes into the airways including mucus hyperproduction, chronic airway inflammation and microbiome dysbiosis. Hence, smoking may induce airway remodelling resulting in fixed airflow limitation (23–27). The risk is accelerated especially in case of positive family history for atopy (28).

Epidemiologically, smoking is highly prevalent among young adults (29) and adult patients with asthma. Unfortunately, tobacco consumption is important in asthma induction not only for the smokers, but also for the unborn infant of smokers for many years to come (30–34).

Maternal smoking during pregnancy is associated with the cumulative incidence of asthma in offspring between the ages of 31 and 46 years (35).

New phenomena are the use of non-tobacco nicotine in different ways of delivery, such as vapes and packages (chewing bags). These products have also been shown to lead to small airways illness and asthma so a switch to them is unlikely to play any role in asthma prevention (36, 37).

###### 2.1.1.2.2 Gas cookers

Nitrogen dioxide fumes from gas cookers is associated with increased asthma with a summary odds ratio of 1.32 [95% confidential interval (CI) 1.18–1.48] (38).

###### 2.1.1.2.3 Other pollutants

The prevalence of asthma and allergies have increased markedly with modern living (39). It appears that this may in part be due to our exposure to environmental chemicals. Around 91% of the world's population are living in areas where the levels of air pollutants exceed the World Health Organization (WHO) limits. There are many different types of airborne pollution, but simplistically these can be divided into gases and particulate matter. The latter is considered as particularly dangerous as respirable particles can remain airborne over large distances.

Air pollution as a risk factor for asthma can also be considered from the perspective of indoor or outdoor sources of pollution. High numbers of domestic cleaning agents are associated with an increased risk of asthma. A Canadian cohort study noted an association between use of cleaning agents early in life and risk of childhood wheeze and asthma at age 3 years (40). Further evidence shows that this association holds across age groups (41). Likely mechanisms are (a) that chemicals increase epithelial permeability and thus allow allergens and pathogens to access the immune cells within the underlying mucosa and (b) that combination with, or altering the structure of, allergens may increase their allergenicity (27). Subsequently, pro-inflammatory immune responses and complex interactions with structural cells and sensory nerves cause chronic airway inflammation (42).

#### 2.1.2 Obesity

Obesity is becoming increasingly common, and it is associated with an increased risk of asthma (43). There are several different mechanisms through which obesity could predispose to or worsen asthma. Obesity may induce pro-inflammatory changes such as increased production of adipokines and pro-inflammatory cytokines, e.g., IL-6 and TNF-α, by adipose tissue. These cytokines cause a low-grade systemic inflammatory response and can induce a non-eosinophilic or neutrophilic airway inflammatory response (44, 45).

Obesity is also associated with co-morbidities such as depression and gastroesophageal reflux disease which can further worsen the clinical manifestations of asthma (43, 46).

When obesity complicates existing asthma, patients report increased symptoms, poorer asthma control, more frequent exacerbations and a poorer quality of life, whereas weight loss increases asthma control. Recent randomized controlled trial (RCT) studies have shown an association between weight-loss and asthma control, as well as increased QoL (47, 48). However, whether weight loss can prevent asthma development is an open question (49).

#### 2.1.3 Psychological factors

Recent data suggests not only a bidirectional association between mood and anxiety disorders and asthma but that asthma can predispose to neurodegeneration, another reason for its prevention (50).

Mendelian randomization studies, which distinguish between association and causation, show a causal relationship between major depressive disorder and asthma with a relative risk of 1.23 (1.13–1.33) (51).

## 3 Allergic asthma

Allergic asthma often begins in childhood as part of a collection of atopic disorders (atopic dermatitis, food allergy, allergic rhinitis) mediated by IgE, the allergy antibody. There is often a family history of allergic disease with multiple associated genes predisposing to allergy and to asthma (52, 53). However environmental factors are also relevant (54, 55).

The Allergic March is one recognised pattern starting in infancy with atopic dermatitis, with subsequent development of asthma and allergic rhinitis (AR). In a recent meta-analysis, some 30% of children with atopic dermatitis (AD) also have asthma, with a figure of 60% in severe AD (56). There is emerging evidence that AD can be predicted through biomarkers measured in non-invasively collected skin tape strips (57), and it is possible that these infants with type 2 skewed skin immune response may also have a higher risk of subsequent asthma development.

Other patterns associated with asthma development may occur, one of which is initial AR, starting in childhood or young adulthood, with subsequent development of asthma (58). Allergic asthma can also begin in adult life. Sometimes this is related to occupational exposures (59, 60).

### 3.1 Concept of pre-asthma

Pre- asthma could be defined as a state in which the subject is experiencing mild, probably intermittent lower airways inflammation/ hypersensitivity which is insufficient to cause symptoms, but which is capable of progression to asthma. Therefore in children with AD or AR or both there is the possibility of identifying those most liable to develop asthma. These are groups where pre-asthma could possibly be identified using cohort studies and sensitive airway assays, such as FeNO, sputum eosinophils or bronchial hyper-reactivity.

Table 1 lists major known risk factors for allergic asthma, some of which could serve to identify those at risk of pre-asthma.

**Table 1:** Risk factors for allergic asthma.

A family history of asthma, allergies or atopic dermatitis increases the risk of childhood asthma
Certain genes are also associated with an increased risk of developing asthma. These include genes for:
1. IL-4 (Interleukin 4): This gene is involved in the regulation of immune responses (61).
2. IL-13 (Interleukin 13): This gene is also involved in immune system regulation (62, 63).
3. FCER1B (Fc receptor, IgE, high affinity I, beta polypeptide): This gene is involved in the production of IgE antibodies, which play a role in allergic reactions (64).
4. ADAM33 (A Disintegrin and Metalloproteinase domain-containing protein 33): This gene is involved in the development of airways remodeling (65).
5. GSTP1 (Glutathione S-transferase P1): This gene is involved in the detoxification of environmental pollutants, variations in it have been associated with an increased risk of asthma in children exposed to tobacco smoke (66).
6. TLR4 (Toll-like receptor 4): This gene is involved in the recognition of pathogens by the immune system (67).
7. Orosomucoid: a protein that is produced by the liver and is involved in immune system regulation (68, 69).
8. G-Protein-coupled receptor (70).
9. Neuropeptide S Receptor 1 (NPSR1) and Retinoid Acid Receptor-Related Orphan 76 Receptor Alpha (RORA) are associated with nocturnal asthma (71).
10. Based on a large recent GWAS ORMDL3 and GSDMB, IL33 and IL1RL1, IL18R1, SMAD3, and IL2RB provide a focus in the search for more effective therapies for asthma (72).
11. Filaggrin (FLG) mutations which increase epithelial permeability (73)
Environmental factors
1. Birth by Caesarean section (74–78).
2. Low birth weight: Children who are born with a low birth weight or who are born prematurely are at an increased risk of developing asthma (30).
3. Exposure to tobacco smoke, air pollution, dust mites, pet dander, and moulds (79, 80).
4. Respiratory infections: Viral respiratory infections, particularly Rhinovirus C and Respiratory Syncytial Virus (RSV), can increase the risk of developing asthma in children (81–86).
5. Allergies: Children with allergies to certain foods, such as cow's milk, eggs, and peanuts, as well as airborne allergens, such as pollen and dust mites, are at an increased risk of developing asthma. Atopic dermatitis (eczema) and Allergic rhinitis can be precursors of asthma (87–90).
6. Obesity: Obesity in childhood has been associated with an increased risk of asthma (91).
7. Gender: Boys are more likely than girls to develop asthma before puberty, but after puberty, the gender difference disappears (92).
8. Ethnicity: Children from certain ethnic groups, such as African-American and Puerto Rican, are at an increased risk of developing asthma (93).
9. Maternal smoking: Maternal smoking during pregnancy and after birth (79, 80).
10. Stress: Stress in pregnancy or chronic stress in childhood may contribute to the development of asthma (46, 91).

These are potential, usually based on associations, but not conclusive or shown to be causative. This is one important reason that several potential interventions are not part of asthma guidelines, e.g., obesity, diet, gastric acid suppression and antibiotic use.

Both genetic predisposition and environmental factors and triggers are implicated in asthma (94). There is interaction between them, for example in children who grow up on a farm and consume raw milk, the protective effect of raw milk is enhanced with CUD14/1721 A allele than with the homozygous G allele (95).

## 4 Prevention of childhood asthma

A risk level for future asthma could be explored. Persons with no predisposing factors could be considered as risk level 0, those with a family history only or one risk factor from Table 1 as risk level 1, and persons with both a predisposing factor and a family history of asthma level 2. A family history of asthma plus two or more risk factors, such as existing FA, AD or AR could be level 3. If there is evidence of lower airway inflammation they could be graded as level 4. Examination of existing clinical data from cohort studies could be mined to validate the appropriateness or applicability of this classification. In fact some such studies already exist (96). Development of childhood asthma prediction models using machine learning approaches (97). The latter improved on regression-based model predictions by utilizing machine- learning approaches.

If a classification proves sufficiently clinically valid, then medical interventions to endangered subjects should then be devised according to level of risk.

Several factors could be addressed, both pre- and post-natally. Genes cannot be altered as yet, but their identification could suggest possible protective environmental measures, such as altering skin pH in AD which reduces asthma development in AD mice (53, 98).

Table 2 shows possible routes of asthma prevention. Many sensible measures, such as avoidance of pollution and good diet are suitable for use at a population level, i.e., primary prevention of asthma in those at all levels, rather than a secondary prevention in those more predisposed to develop asthma. Each area is expanded in the text.

**Table 2:** Factors influencing asthma development and possible strategies for its prevention.

Factors	Possible prevention strategy	Factors	Possible prevention strategy
Maternal		Infant	
Genetics	No	Genetics	No
Obesity *Level 0* Stress *Level 0*	Prevention of obesity in pregnancy may reduce risk of infants developing asthma. In case weight loss in pregnancy is needed it should be always guided by a specialist. Counselling fertile women re weight- research needed Psychological factors such as stress and depression increase asthma risk Avoid/reduce stress	Breastfeeding *Level 0* Microbiome Food introduction *Level 1–4* Omega 3 *Level 1+* Obesity *Level 0*	Breastfeeding gives protection from asthma/wheeze Encourage and support breastfeeding In children and infants establishing a stable microbiome is essential for immune homeostasis, nevertheless most probiotics failed the primary outcome of preventing asthma. Research needed Early food introduction reduces risk of food allergy in susceptible children. Increased food diversity post-natally reduces the risk of developing asthma Dietary advice A higher dietary intake of omega-3 fatty acids in childhood may reduce the risk of developing subsequent asthma but only in children carrying a common gene variant in the fatty acid desaturase (FADS) gene Dietary advice, research needed Increases asthma risk. Avoid ultra- processed foods Research -based dietary advice
Medication use *Level 1–4*	Avoiding antibiotics and acid suppressants during pregnancy may reduce risk of asthma. Better antibiotic stewardship Advice to pharmacists/GPs/public Research needed	Farm living *Level 0* Level 0 Level1–4	Children who grew up on a farm with animals have a reduced risk of developing asthma. Unpasteurised milk also beneficial. Contact with nature BCG vaccination—investigate optimal timing ? specific bacterial product(s) later Research needed
Smoking *Level 0*	Avoiding maternal smoking and passive smoke avoidance reduces the risk of children developing asthma Advice to all fertile women and their families	Allergen avoidance	No supportive data
Diet *Level 0*	Cooked vegetables and yoghurt ? Mediterranean Advice to all fertile women	Environment *Level 0*	Avoidance of smoke/ gases/ pollutants/ chemicals which damage epithelia, both outdoors and indoors Advice to parents/family/carers
Delivery *Level 0*	Caesarean section increases asthma risk Vaginal delivery where possible	RSV, rhinoviruses *Level 0*	Severe respiratory illness may be associated with subsequent asthma. The risk is greatest for young children and may be increased by prematurity. Vaccination may be helpful Monitor effect of new RSV vaccine
	* *	Antibiotics *Level 0*	Avoid unnecessary use Better antibiotic stewardship
		Atopic dermatitis *Level 1–4+*	Consider dupilumab for severe AD Monitor effect on asthma development-research needed
	* *	Allergic rhinitis *Level 1–4*	Allergen immunotherapy for AR can reduce the incidence of new-onset asthma and new sensitizations, as well as reducing the burden of asthma medication in those with established disease Identify children most likely to benefit- more research needed
		Holo BG Level 1–4	Research needed

Major known factors predisposing to asthma are listed, together with suggested measures to reduce its incidence. Level 0 means that this would be applicable at the level of the whole population. Level 1 or higher for those at greater risk, particularly for those at level 4, those who have pre- asthma, defined here as a state in which the subject is experiencing mild, probably intermittent lower airways inflammation which is insufficient to cause symptoms, but which is capable of progression to asthma.

HDM SLIT, House Dust Mite Sublingual Immunotherapy; RSV, Respiratory Syncytial Virus; SCIT, Subcutaneous Immunotherapy; SLIT, Sublingual Immunotherapy.

### 4.1 Maternal factors

#### 4.1.1 Obesity

Maternal obesity increases the risk of developing asthma in offspring. In mothers who were obese, each added 1 kg/m^2^ was associated with a 2–3% increased risk of the infants developing asthma at an age between 14 months and 16 years (99). However, the GINA guidelines, an initiative with the goals of disseminating information about asthma management, and providing a mechanism to translate scientific evidence into improved asthma care, do not recommend unguided weight loss in pregnancy (14).

#### 4.1.2 Diet and microbiome

A healthy gut microbiome in early infancy can be influenced by maternal diet during pregnancy as well as the maternal environment. It can affect the development of immunity and alter the predisposition to later immune- mediated diseases, such as asthma (100). The protective effect against allergies of having older siblings may be mediated via the gut microbiota (101).

Whereas there is little evidence in favour of dietary allergen avoidance in pregnancy (14), non—allergenic food intake during pregnancy was assessed in a systematic review of studies investigating any association between it and all allergic outcomes in the offspring. A Mediterranean diet rich in vegetables, legumes and high on fish/fatty fish and Vitamin D appeared protective from developing allergies and asthma. Because of the heterogeneity of the studies and lack of clarity on intervals and measurement of intake no clear recommendations could be made (102, 103).

More comprehensive work in the Healthy Start study (104) was on a cohort of over 1,200 mothers and had a reliable maternal diet index. This index weighted measures of intake of vegetables, yogurt, fried potatoes, rice/grains, red meats, pure fruit juice, and cold cereals. A prenatal diet that included cooked vegetables and yoghurt reduced the risk of allergic disease at 4 years. In adjusted models, a one-unit increase in the index was significantly associated with reduced odds of offspring allergic rhinitis [odds ratio (CI) 0.82 (0.72–0.94)], atopic dermatitis [0.77 (0.69–0.86)], asthma [0.84 (0.74–0.96)], and wheeze [0.80 (0.71–0.90)], but not food allergy [0.84 (0.66–1.08)]. A diet consisting mainly of fried potato, red meat and undiluted fruit juice had an increased risk of allergic disease. As the authors state: “This is the first study that has shown associations between an index of maternal dietary intake during pregnancy and multiple offspring allergic diseases. The results give hope for prevention of allergic diseases *in utero*.”

#### 4.1.3 Gastric acid suppressants

The sensitization capacity of acid suppressing drugs was confirmed in a recent study performed as an investigation of three human databases in Sweden, which found a positive correlation with such medication taken by the mother during pregnancy and the induction of allergy and asthma in the child (105).

#### 4.1.4 Maternal antibiotic use

Pre-natal antibiotics have been shown to lead to increased risk for asthma. In a cohort of 213,661 mothers from 1996 to 2012, of the 38.6% of mothers who received antibiotics 10.8% of their infants developed asthma. Maternal antibiotics nine months prior to pregnancy and nine months postpartum also gave an increased risk of developing asthma (106).

These results could not be confirmed in a similar study in a Danish population (107). From 2005 to 2011, 22.3% of 407,804 women received antibiotics during pregnancy Antibiotic use during pregnancy was associated with childhood asthma in cohort analyses (HR 1.21, 95% CI 1.18–1.24), but not in sibling analyses (HR 0.96, 95% CI 0.90–1.03). This suggests that previous studies showing increased risk may have had confounders such as differences in genetics and in history of environmental exposure.

#### 4.1.5 Smoking

As mentioned earlier, maternal smoking carries a very high risk of children developing asthma. In a review of 70 publications, citing 71 studies, the risk of developing wheeze with maternal smoking was 52% with a 20% increased risk of developing asthma between 5 and 18 years. There was an increased risk for maternal passive smoking as well as for household smoke. Longitudinal studies show that postnatal altered indices of airway function are not only a risk factor for subsequent asthma or wheezing illness development but also predict adult airway dimensions. Vitamin C supplementation to pregnant smokers might ameliorate the effects of *in utero* smoke on offspring lung function. Interventions in well-phenotyped longitudinal birth cohorts with early airway function assessments monitored through to adulthood are needed (79, 80).

### 4.2 Perinatal factors

#### 4.2.1 Birth by caesarean section

Birth by caesarean section (CS) is associated with adverse immune effects including predisposition to infections, allergies, and inflammatory disorders, probably related to failure to collect the vaginal microbiota. The lower success of breastfeeding after CS adds to alterations in normal microbiota development (74–77).

Caesarean section was associated with an increased risk of asthma at age 8 in a birth cohort study of 2,917 children (OR 1.79; 95% CI 1.27–2.51). The risk was higher with two allergic parents: OR 2.91; 95% CI 1.20–7.05; compared to one: OR 1.86; 95% CI 1.12–3.09). In those with non-allergic parents the OR was 1.36; 95% CI 0.77–2.42) (78).

### 4.3 Post natal factors (INFANT)

#### 4.3.1 Breastfeeding

Breastfeeding is protective of asthma/wheeze from 0 to 2 years at which time the protective effect seems to wane (92). There was however a protective effect from developing asthma from 5 to 18 years based on meta- analysis by Lodge (108).

Breastfeeding data in weaker- designed studies might have a recall bias or difference in breastfeeding data might be attributable to the duration of breastfeeding. Breastfeeding helps establish the infant's gut microbiome that takes up to 1,000 days to stabilize (Pregnancy to 2 years). The Oligosaccharides (HMO), IgA, protein, free amino acids, vitamins, cytokines and antibacterial peptides in breastmilk play a role not only in establishing the gut microbiome of infants as well as their innate and adaptive immune system. Breastfed infants have 90% *Bifidobacteria* colonisation and formula fed infants have 40% *Bifidobacteria* and 60% *Lactobacillus* (109).

#### 4.3.2 Dietary

Increased food diversity post-natally in the first year of life had an inverse relationship to developing asthma and with each new food introduced there was a relative risk reduction of developing asthma with a dose-response effect [adjusted odds ratio with each additional food item introduced, 0.74 (95% CI, 0.61–0.89)]. A similar effect was observed for food allergy and food sensitization. Non- pasteurised milk, butter, and yoghurt as well as fruit and vegetables gave the greatest protection. Increased dietary diversity was associated with an increase expression of Foxp3 on T regulatory cells, indicating a possible inhibition of class switch to IgE at 6 years (87, 88).

The timing of food introduction has been shown to have a crucial effect upon the development of food allergies in predisposed infants (89, 90). Since food allergy predisposes to asthma development this is an important area for identification and management of those at risk, i.e., those with pre- asthma.

##### 4.3.2.1 Supplementation

###### 4.3.2.1.1 Vitamin D

The association between vitamin D and development of allergy is contradictory with some evidence of a U-shaped curveA publication reviewed pre- natal vitamin D supplementation and concluded that it probably prevented transient wheezing, but not allergic asthma (110). A recent large Japanese study of almost 74,000 mother—infant pairs showed no association between maternal vitamin D levels and asthma or atopic dermatitis at age 3. However there was an inverse relationship between maternal dietary vitamin D intake and the risk of developing allergic rhinitis symptoms at the age of 3 years (111). Since rhinitis predisposes to asthma further follow up is necessary. Large, well-planned randomized controlled trials on vitamin D supplementation are needed.

###### 4.3.2.1.2 Omega 3 fatty acids

A higher dietary intake of long chain omega-3 fatty acids in childhood may reduce the risk of developing subsequent asthma, but only in children carrying a common gene variant in the fatty acid desaturase (FADS) gene which is associated with lower blood levels of long chain omega-3 fatty acids (112).

###### 4.3.2.1.3 Probiotics

Probiotic supplementation may be the next step in correcting gut dysbiosis and preventing asthma. Heterogeneity of study design as well as of probiotic strains used contribute to the complexity of evaluating the available data. Most probiotics evaluated failed the primary outcome of preventing asthma. Individual probiotics seem to have different effects, some more beneficial than others. *Lactobacillus rhamnosus* GG was studied in infants 6–24 months for 6 months. The supplement had no effect on development of asthma or eczema. The treatment group had mild effect on decreased sensitisations and less severe asthma, 6 months after discontinuation of the supplementation (113). The answer may be to use pre- or synbiotics (114). Studies are needed.

###### 4.3.2.1.4 Bovine holo-beta-lactoglobulin

Micronutritional supplementation with a holoBLG-based food for special medical purposes (FSMP) lozenge alleviates allergic symptoms in BALB/c mice, imitating the protective effect of farm living (vide infra) (115, 116). It appears to act by inserting complexed iron into immune cells. Clinical studies have shown that holo beta-lactoglobulin (holoBLG) can restore micronutritional deficits in atopic immune cells and alleviate allergic symptoms in a completely allergen-nonspecific manner (117). A recent study demonstrates protection against cat challenge in cat allergic human subjects (118). The effects of providing such micronutrition on a large scale to pre-atopic children is unknown, as yet.

#### 4.3.3 Farm living

Microbiota colonise our skin, gut and respiratory system and play a regulatory role between inflammation and immune tolerance. In children and infants establishing a stable microbiome is essential for immune homeostasis. Children who grew up on a farm have a risk reduction of developing asthma between 32% and 78%. In the GABRIEL and PARSIFAL studies, children who grew up on a farm had an increased exposure to endotoxins and an inverse relationship to asthma and allergic rhinitis (119, 120).

The protective effect in pre-school -aged children in the Genetic and Environmental Causes of Asthma in the European Community (GABRIEL) study found an inverse relationship of allergy to exposure to cows that live in shed and their fodder. The timing of exposure has an enhanced protective effect with pre- natal exposure to farm animals offering protection from developing atopic dermatitis in infancy and from asthma and allergic rhinitis at school age (121, 122).

Consuming dairy products that are not pasteurised had an inverse relationship with developing respiratory allergies, hay fever and asthma. This relationship was found in four large European studies. The increased levels of whey in farm milk might be the explanation as there was an inverse relationship between bovine serum albumin (BSA), lactoglobulin and lactalbumin. Higher levels of polyunsaturated fats and TGF -beta in farm milk were inversely related to asthma as well as positively to increased regulatory *T*-cell levels in children at age six.

Endotoxin—bearing dust from homes of Amish families, who farm traditionally with animals rather than machinery, administered nasally to mice inhibited eosinophilia and airway hyperreactivity, unlike dust from genetically similar Huttite communities who use modern farming methods (123). The protective effects failed in mice deficient in innate immune signalling molecules MyD88 and Trif.

If there are particular organisms related to this farm protective effect it might be possible to use them, or molecules derived from them, in future to prevent allergic diseases, including asthma.

#### 4.3.4 Infections

##### 4.3.4.1 Viral

Respiratory viruses are known to precipitate asthma attacks, but also appear to invoke the development of asthma (81). Although 30%–50% of children will wheeze before 1 year, and 30%–40% will have recurrent wheezing, the prevalence of asthma is 5%–10% in children. Respiratory syncytial virus (RSV) is the main cause of bronchiolitis; Rhinovirus (RV) is usually detected in wheezing children thereafter. Severe respiratory illness with either virus is associated with subsequent asthma. The risk is greatest for young children who wheeze with RV infections. They could be labelled as having pre- asthma. Better knowledge of factors promoting more severe viral illnesses might lead to new prevention strategies, possibly reducing the subsequent risk for asthma**.**

###### 4.3.4.1.1 RSV

RSV typically causes bronchiolitis in the 3–6 month age group, by the age of 2 most children have had RSV. Risk factors for severe RSV infection is age below 3 months, chronic lung disease, prematurity and immune deficiency. RSV infection is associated with recurrent wheezing episodes in childhood. Infants who avoided infection with respiratory syncytial virus, or RSV, during the first year of life had a 26% lower risk of developing asthma by age 5 (82).

The anti-RSV monoclonal antibody Palivizumab decreases the risk of severe RSV-induced illness and subsequent recurrent wheeze, but not asthma. In a systematic review live attenuated RSV vaccine in the 6–24 month-old group resulted in a 4-fold increase in anti-F antibodies in 90%–95% of children. Nirsevimab, a long-acting monoclonal antibody that lasts 5 months, reduces hospitalisation and severe disease in healthy premature infants (83). Whether RSV vaccination prevents asthma development is as yet unknown.

Indirect immunological protection is also being explored, reducing the age for the measles vaccine from 6 to 4 months to protect high risk infants (84, 85).

###### 4.3.4.1.2 Rhinovirus

RV-A and RV-C are associated with more viral asthma exacerbations than RV-B. RV-C is associated with more hospitalisations. The effects of viral exacerbations in allergic asthma are dose related. Higher allergen exposure and higher IgE levels lead to more severe viral exacerbations of allergic asthma. Human rhinovirus type C receptor CDHR3 polymorphisms have been shown to affect receptor epithelial expression, activation, and asthma development and exacerbation severity in children (86). Anti-inflammatory treatments have efficacy for RV-induced wheezing (81) which is usually time -limited, not progressing to asthma.

##### 4.3.4.2 Bacterial

Neonates colonized in the hypopharyngeal region with *S. pneumoniae*, *H. influenzae*, or *M. catarrhalis*, or with a combination of these organisms, are at increased risk for recurrent wheeze and asthma early in life (124).

Acute wheezy episodes in young children were significantly associated with bacterial infections, similar to, but independent of, the association with virus infections (125).

#### 4.3.5 Antibiotics

The use of antibiotics in young children does not appear to be protective against asthma, but is rather associated with an increase in wheezing and allergies.

A cross-sectional pre-schooler study in Shanghai, China involved 13,335 questionnaires (response rate: 85.3%). A quarter of the children (*N* = 3,049; 24.1%) had antibiotic exposure in their first year of life. In multivariate logistic regression analyses this had significant associations with higher odds of wheeze (1.44, 1.30–1.60), asthma (1.38, 1.19–1.61), food allergy (1.29, 1.13–1.46), and allergic rhinitis (1.23, 1.07–1.41). These associations differed in children with different individual characteristics (age, sex, family history of atopy, and district) and environments (breastfeeding, home decoration, pet-keeping, and environmental tobacco smoke) (126). The mechanism may relate to effects on the gut microbiome (127). Data in adults also supports an association between macrolide use and asthma in 20–40 years and 40–60 years age groups. For individuals over 60 years old, quinolones were significantly associated with asthma (128).

#### 4.3.6 Vaccination against tuberculosis with *mycobacteria bovis* BCG

The Bacille Calmette-Guérin (BCG) vaccine against tuberculosis also protects children from RSV hospitalisation and mortality as seen in vaccinated vs. non- vaccinated populations (129).

A recent meta-analysis involving twenty studies from 1950 to 2021 with a total of 222,928 participants concludes that *M. bovis* BCG in early life also protects against asthma development (OR 0.77, 95% CI 0.63–0.93), indicating a protective efficacy of 23% against asthma development among vaccinated children. However, early administration of tuberculosis vaccine did not significantly reduce the risk of developing eczema (OR 0.94, 95% CI 0.76–1.16) and rhinitis (OR 0.99, 95% CI 0.81–1.21). The effect of *M. bovis* BCG on asthma prevalence was significant especially in developed countries (OR 0.73, 95% CI 0.58–0.92) (130).

#### 4.3.7 House dust mite sublingual immunotherapy (HDM SLIT)

A small study tried SLIT as a means for primary prevention of allergy in the first year of life. In a blinded pilot study 111 infants less than 12 months, at high risk of atopy based on heredity (>2 first-degree family members with allergic diseases [asthma, allergic rhinoconjunctivitis, eczema or food allergy] but negative SPT responses to HDM, grass pollen, cat, peanut, milk and egg, were given HDM SLIT for 12 months. There was a 16.0% (range 1.7–30.4%) reduction in developing any sensitisation to common allergens. There was no statistical difference in the other primary outcome of HDM sensitisation, nor in allergic disease development. Follow up and future studies are needed to better evaluate this as an intervention (131).

#### 4.4.1 Emollients to prevent AD (and hence, possibly asthma)

Early AD is associated with asthma at school age. The broken skin of AD may represent a site for further allergen sensitization and disease progression. However, wheezing occurs in many of these children before or with the onset of AD and a marked loss in lung function is often noted, suggesting a distinct phenotype rather than a progression from AD to asthma (132).

However, prevention of AD is a worthwhile goal and any effect upon later asthma could be noted. Studies using emollients to prevent AD were initially encouraging, with a 50% reduction in atopic dermatitis development (133). However, two large, randomized trials (134, 135) have failed to confirm this observation and routine emollient use is not generally recommended. Avoidance of harsh detergent soaps and allergenic materials on the skin of infants is advised.

#### 4.4.2 Other AD treatments

A meta-analysis of the database on dupilumab in AD from 12 clinical trials in AD showed a 37% overall reduction of the risk of development of new allergies, this rate was 54% when IgE was included. The best response was reached in patients under 18 years with white ethnicity background, early onset of AD (at age <2 years), severe AD, and asthma at baseline. Further data is needed to decide whether asthma incidence can be reduced by dupilumab use in severe AD (93).

### 4.5 Upper airway disease

Both allergic and late onset asthma have a high prevalence of upper airway disease. Over three quarters of teenage asthma subjects also suffer from rhinitis (136). A European longitudinal study showed that both AR and NAR carried an increased risk of asthma, with that of AR being over threefold (136).

This has been confirmed in younger children where AR until age 5 years predicted wheezing development between the ages of 5 and 13 years, with an adjusted relative risk of 3.82 (*P* < .001). This association was not related to sensitization type or severity, nor to AD during the first 2 years of life. Among the 1,314 children studied, 41.5% of all new cases of wheezing occurred among those with preceding AR (137).

This may offer an opportunity for prevention. Although allergic rhinitis requires effective treatment on its own merits, it could be helpful to identify those children most likely to progress to asthma and ensure that they are well—controlled. Those with early onset, severe and persistent allergic rhinitis symptoms may be at a higher risk of developing asthma, especially if other allergic conditions, such as food allergy and atopic dermatitis are present. A Korean study suggests that it is those with a high atopic burden and impaired lung function who are at higher risk of asthma (138, 139). Nasal challenge results in eosinophilic inflammation to the bronchi and vice versa (140). AR causes bronchial hyperreactivity (141), so perhaps reducing allergen exposure and controlling nasal inflammation well could reduce asthma development?

#### 4.5.1 AR and asthma prevention

Pharmacotherapy for AR can improve asthma control (142) but there is no good evidence for its preventing asthma development.

##### 4.5.1.1 Allergen immunotherapy

Recent cohort data shows that approx. 75% of children with pollen-AR at 4 or 8 years had persistent disease up to 24 years, and 30% developed asthma (143). Further investigation to determine any identifiable pre- asthma characteristics in this cohort would be helpful.

There is considerable retrospective, observational data to suggest that allergen immunotherapy given for allergic rhinitis can reduce the incidence of new-onset asthma, as well as reducing the burden of asthma medication in those with established disease. Studies of prescription databases in France and Germany have demonstrated this for grass and birch pollen immunotherapy (144–146).

A study of a national health insurance database in Germany provided similar results for a broader range of both seasonal and perennial allergens (147), with similar results for both seasonal and perennial allergens. The overall incidence of asthma in these cohorts was in the region of 1%–2% over the course of the timeframe studied, with a relative risk of 0.6–0.7 in individuals receiving immunotherapy compared to those not. Treatment for at least 3 years appeared to give a greater effect (136). Of note, a further study using German national health insurance data found a slight increase in the chance of new onset asthma in the group receiving immunotherapy, despite seeing reductions in asthma medication use and frequency of asthma exacerbations in the immunotherapy group (148).

A number of prospective, randomised controlled trials have also investigated the effect of immunotherapy on asthma prevention. A European Academy of Allergy and Clinical Immunology (EAACI) systemic review and meta-analysis reviewed these and concluded there was evidence for reduction of risk of new onset asthma in the short term (up to 2 years after completion of treatment) (149). This was evident only for both sublingual and subcutaneous immunotherapy, but only in individuals younger than 18 and only with pollen immunotherapies. They were unable to conclude that the effect persisted in the longer term (beyond 2 years). A study has suggested an effect up to 10 years, though with high risk of bias (150–152). The Grazax Asthma Prevention (GAP) study looked at the preventative effect of 3 years of grass pollen sublingual immunotherapy tablets vs. placebo on new incidence of asthma in children aged 5–12 (153). Whilst the primary outcome—symptomatic asthma with demonstrable beta-agonist airflow reversibility—was not significantly different in the active arm, secondary outcomes were significant and included fewer asthma symptoms and less asthma medication use in the active group. The effect was greatest in younger children.

Overall, data for a protective effect on asthma development is most robust for pollen allergens and in children and for 2 years after completion of treatment. The effects may persist for longer, be applicable to perennial allergens, and hold true in adults (154), but high-quality confirmatory data is required as well as identification of those individual most likely to benefit.

### 4.6 Other measures

Neither prophylactic use of inhaled corticosteroids nor of the antihistamine cetirizine showed any protective effect against asthma development in clinical trials. Non- interventional longitudinal studies do not suggest a role for allergen avoidance in early life (155, 156).

## 5 Discussion

The ten- year Finnish allergy programme was associated with a levelling off of the prevalence of allergic asthma and rhinitis. This suggests that their primary causes are environmental and that both can be prevented by nature- relatedness, active mobility, and sustainable diet (157).

Currently costs of asthma are high to individuals, healthcare systems and to society, often involving lifelong treatment, once initiated. In the United Kingdom (UK), 1.1 million children (1 in 11) are currently receiving treatment for asthma and most adult asthma begins in childhood. The National Health Service (NHS) spends around £1 billion a year treating and caring for people with asthma (158). Reduction in incidence would improve quality of life and would likely be cost effective. Primary prevention measures have been suggested previously. Have we advanced any further than the following recommendations given in 2015?

…“ public health efforts should remain focused on measures with the potential to improve lung and general health, such as: reducing tobacco smoking and environmental tobacco smoke exposure; reducing indoor and outdoor air pollution and occupational exposures; reducing childhood obesity and encouraging a diet high in vegetables and fruit; improving feto-maternal health; encouraging breastfeeding; promoting childhood vaccinations; and reducing social inequalities.”

The answer is yes. The better understanding of the role of ultra processed foods in causing obesity would suggest that avoidance of these should be included, together with increased outdoor time in nature, allowing exposure to archaeo-organisms such as mycobacteria (91). These are sensible at a population level, as are warnings against unnecessary use of antibiotics and acid- suppressants in pregnancy. Reduction of the currently high rate of caesarean sections is another target which could decrease asthma incidence.

Other areas worth investigation relate to micro- organisms. Some observations in a previous EUFOREA paper (159) were that the one allergen still to be avoided was latex in children with spina bifida and that there were anti- wheezing effects of bacterial mucosal vaccines in recurrently wheezing pre-school children (160). Although not all early wheezing is asthma and the children in this study were non- atopic, it does show an effect of bacterial molecules upon innate immunity. Detailed analysis, possible with UK health records, into BCG and asthma occurrence might reveal an optimal timing for this to be given, either to the population or to those most at risk. The long-term results of RSV vaccination once available may show a reduction in asthma incidence. Prevention of rhinovirus infections has long been a goal which still appears unreachable. Farm dust may provide therapeutic molecules.

Supplementation with Beta Holo Lactoglobulin could be a promising intervention but needs more research.

Targeted therapy for those most at risk, i.e., those with pre-asthma, needs to be more specific. For pre-diabetes, levels of hyperglycaemia and HbA1c are used (161), though there is no universally agreed definition. Identification of likely pre-asthmatics could be as suggested earlier via family history, existing allergy to food, atopic dermatitis or upper airway disease, supplemented by tests for early lower airway involvement. No further test has yet been applied, but possible tests could include: lung function testing (including reversibility, impulse oscillometry and bronchial hyperresponsiveness), FENO, eosinophil counts in nasal secretions or sputum. Inclusion of these markers in future clinical trials would be helpful in reaching a definitive pre-asthma diagnosis. In children who already wheeze both FENO and specific IgE measured at 4 years were associated with wheezing and asthma at 8 years. Both tests also remained significant predictors after mutual adjustment and adjustment for clinical history: OR on wheezing at 8 years for FE(NO) (9) log-scale, per IQR) 1.6 (95% CI 1.1–2.2) and for specific IgE 2.8 (95% CI 1.9–4.1) (162). It may be that different definitions are needed for different therapeutic approaches: for example, severe AD carries a 60% risk, AR a threefold one. Severe sufferers with either of these could be regarded as likely to be pre-asthmatic and treated accordingly with careful characterisation, follow up and evaluation for more definitive identification of factors leading to progression. On a population level the new tool of AI could be used to trawl large healthcare databases for markers in subjects who developed asthma.

Allergen immunotherapy (AIT) for AR shows promise in asthma reduction, even in real life studies. Some allergens, such as those from animals and HDM are more highly associated with asthma than are pollens. In the European Community Respiratory Health Survey the overall attributable fraction (AF) of asthma symptoms caused by atopy was 45% with a physician diagnosis of asthma. The AF for atopy was significantly correlated with sensitization to house dust mite (*r* = 0.64). The percentage of asthma attributable to HDM sensitization was 18.2% in the overall population and 12%–48% in various study centres in France (163). Sublingual is significantly safer and more convenient than subcutaneous immunotherapy (164). The new HDM SLIT tablet is safe and well tolerated in adults (165). A study on safety and effectiveness in AR in children, where it is most likely to decrease subsequent asthma, has just completed (166). Studies are needed to see if its use reduces progression to asthma and, if so, on identifying those likely to respond to such treatment. Recent evidence suggests that AIT may to some extent repair epithelial barrier function (167). Upper airway disease needs to be taken more seriously with accurate diagnosis of AR, since it is likely that, if effectively treated early, some asthma may be prevented. EUFOREA is active in this regard with both paediatric and adult guidelines for AR management (168, 169) and suggestions for increased use of AIT in the recent summit paper (170).

Similarly, the use of dupilumab in atopic eczema needs to be closely monitored for any anti- asthma effect in real life. Employing artificial intelligence to healthcare databases and those from clinical trials might provide useful information using big data.

Identification of subjects most at risk of asthma (pre- asthma) followed by therapeutic intervention is becoming a possibility, but requires further research.

## Data Availability

The raw data supporting the conclusions of this article will be made available by the authors, without undue reservation.
